# Rapid and Effective Removal of Cu^2+^ from Aqueous Solution Using Novel Chitosan and Laponite-Based Nanocomposite as Adsorbent

**DOI:** 10.3390/polym9010005

**Published:** 2016-12-27

**Authors:** Jie Cao, Han Cao, Yuejun Zhu, Shanshan Wang, Dingwei Qian, Guodong Chen, Mingbo Sun, Weian Huang

**Affiliations:** 1 School of Petroleum Engineering, China University of Petroleum (East China), Qingdao 266580, China; ham_cao@163.com (H.C.); qdw1995@126.com (D.Q.); c17854227168@126.com (G.C.); masterhuang1997@163.com (W.H.); 2 State Key Laboratory of Offshore Oil Exploitation, Beijing 100027, China; zhuyj3@cnooc.com.cn (Y.Z.); wangshsh24@cnooc.com.cn (S.W.); 3 CNOOC Research Institute, Beijing 100027, China

**Keywords:** heavy metal removal, nanoparticle-polymer hybrids, Laponite, biosorbent, rapid adsorption

## Abstract

In this paper, a novel method for preparing nanoparticle-polymer hybrid adsorbent was established. Laponite was dispersed in distilled water to form Laponite nanoparticles. These nanoparticles were pre-adsorbed by 2-acrylamido-2-methylpropane-sulfonic acid (AMPS) to improve their dispersion stability in chitosan solution. The nanoparticle-polymer hybrid adsorbent was prepared by copolymerization of chitosan, acrylamide, acrylic acid, AMPS, and Laponite nanoparticles. Four adsorbents were obtained and characterized by Fourier transform infrared spectroscopy (FTIR), scanning electron microscopy (SEM), and Brunauer-Emmett-Teller adsorption (BET). Additionally, the uptake capacities of Cu^2+^ using different samples were studied. Compared to the adsorbent without chitosan and Laponite components, the maximum uptake of the hybrid adsorbent increased from 0.58 to 1.28 mmol·g^−1^ and the adsorption equilibrium time of it decreased from more than 75 min to less than 35 min, which indicated that the addition of chitosan and Laponite could greatly increase the adsorption rate and capacity of polymer adsorbent. The effects of different experimental parameters—such as initial pH, temperature, and equilibrium Cu^2+^ concentration—on the adsorption capacities were studied. Desorption study indicated that this hybrid adsorbent was easy to be regenerated.

## 1. Introduction

Water pollution of heavy metal ions is now increasingly serious with the development of industry. Heavy metal ions such as copper, cadmium, chromium, lead, and mercury ions are highly dangerous to human beings as well as the environment, even at low concentration levels in water. These heavy metal ions tend to accumulate in the tissues of living organisms, whether in plants or animals, and cause many problems such as inhibiting natural growth and changes in the structure and function of cells resulting in disease and even cell death [1,2,3,4,5]. For this reason, the treatment of toxic heavy metal ion pollution in water has received an extensive concern and become a hot topic in environmental research. Various methods such as coagulation/precipitation, ion-exchange, adsorption, oxidation/reduction, electrolysis, solvent extraction, and membrane filtration have been developed to remove heavy metal ions from contaminated water [6,7,8,9,10,11,12,13,14]. Among these methods, the adsorption process seems to be the most versatile and effective method if combined with appropriate regeneration steps [15,16,17,18]. As a result, different types of modified/unmodified biopolymer-based adsorbents have been fabricated and used for heavy metal ion removal [19,20,21,22,23]. Although these polymers are inexpensive, environmentally friendly, non-toxic, and biodegradable in nature, but their high solubility, poor metal-chelation capability, low surface area, and lack of ionic charges on the polymer surface restrict their extensive utility as an adsorbent [24,25]. Recent advances in nanoscale science and engineering suggest that nanostructured materials and particles exhibit good adsorption efficiency and rapid removal properties especially due to their high surface area and great amount of active sites for interaction with heavy metal ions [26,27,28].

Laponite, a synthetic clay, has alumina silicate mineral layers with negative charges that make for an excellent cationic adsorption property due to its significant surface area [29,30]. The individual particles of Laponite are exfoliated in aqueous solution and the size of a single Laponite crystal is roughly 25 nm in diameter and 0.92 nm thick [31,32]. However, due to their sensitivity to ions, Laponite nanoparticles tend to cause aggregation and gelation in an ionic environment. To avoid its dispersion problem, Laponite can be pre-adsorbed by 2-acrylamido-2-methylpropanesulfonic acid (AMPS) before further mixture with ionic chemicals [33]. Chitosan is usually derived from the deacetylation of chitin from the shells of shrimp, crab, and other arthropods and has been widely used in pharmaceutical industries for drug delivery [34,35]. Chitosan is a polycationic polymer consisting of d-glucosamine and *N*-acetyl-d-glucosamine, so it is a multifunctional polymer that has primary and secondary hydroxyl groups, as well as highly reactive amino groups. It is regarded as a useful starting support for adsorption purposes [36,37]. Until now, chitosan-based adsorbents have been used in the removal of copper, lead, cadmium, and mercury ions from aqueous solution [38,39,40].

In this paper, we established a novel chitosan and Laponite-based nanoparticle-polymer hybrid adsorbent. In this adsorbent, there are a lot of functional groups such as –COO^−^, –NH_2_, –OH, –CONH_2_, and –SO_3_^−^, which is conducive to adsorption. Moreover, the nanocomposite structure also improves the adsorption rate and efficiency. As a result, the adsorption kinetics and efficiency of this adsorbent on the removal of Cu^2+^ was studied.

## 2. Materials and Methods

### 2.1. Materials

Chitosan, which has, when measured by viscometry, a molecular weight of 2.4 × 10^5^ g·mol^−1^ and an 85% degree of deacetylation, was supplied by Haidebei Marine Bioengineering Co. (Jinan, China), refined twice by dissolution in a 0.1 mol·L^−1^ HCl solution, filtered, precipitated with ethanol, and finally dried in vacuo at 50 °C for 48 h. Laponite XLG (Mg_5.34_Li_0.66_Si_8_O_20_(OH)_4_Na_0.66_) was provided by Rockwood (Wesel, Germany). Acrylamide (AM, 98%), acrylic acid (AA, 98%), 2-acrylamido-2-methylpropanesulfonic acid (AMPS, 98%), potassium persulfate (KPS, 99%) and *N*,*N*′-methylenebisacrylamide (MBA, 98%) were purchased from Sinopharm Chemical Reagent Co. Ltd. (Shanghai, China). Other chemicals were analytical reagents and used as received.

### 2.2. Preparation of Adsorbent

Chitosan was dissolved in 2 wt % AA solution in order to obtain a concentration of 5 wt %. Laponite XLG was dispersed in deionized water under ultrasonication for 30 min and the concentration is 2 wt %. A certain amount of AMPS was added into the Laponite dispersion and a uniform dispersion was obtained under stirring for 2 h. Other chemicals including AM, chitosan, AA, and MBA were added. After stirring for 3 h, 10 g of KPS solution (0.5 wt %) was added into the monomer system and the polymerization was performed at 70 °C in a nitrogen atmosphere. The product was dried in vacuo at 65 °C for 48 h and sieved to obtain particles between 40 mesh and 20 mesh.

### 2.3. Characterization

FTIR was carried out on a Tensor 27 spectrometer (Bruker, Switzerland) with sample prepared as KBr pellets. The spectra were acquired in the frequency range 4000–400 cm^−1^ at a resolution of 4 cm^−1^ with a total of 16 scans.

The morphologies of the surfaces of dried S-2 and S-4 samples were observed with a scanning electron microscope (JSM-7600F, JEOL, Tokyo, Japan) coupled with an EDS (Energy Dispersive X-ray spectroscopy) system. The surfaces of samples were coated with a thin layer of gold before SEM examinations.

The pore surface area of four samples was determined using a specific surface area and pore analyzer (NOVA 3200e, Quantachrome, Boynton Beach, FL, USA). Prior to adsorption at 77 K, samples were degassed for 24 h. The specific surface area was calculated by the Brunauer-Emmett-Teller equation. The total pore volume was obtained from the amount of nitrogen adsorbed at a relative pressure of about 0.99. The pore size distribution was derived from the desorption isotherms on the basis of the Barrett-Joyner-Halenda (BJH) method. The average pore size *D* (nm) of sample was determined by the following equation:

*D* = 4*V*_total_/*A*_BET_ (1)

where *A*_BET_ (m^2^·g^−1^) is the specific surface area, *V*_total_ (cm^3^·g^−1^) is the total pore volume.

### 2.4. Adsorption Experiments

The removal of Cu^2+^ onto adsorbent was carried out by batch method and the influence of contact time, initial pH, temperature, and equilibrium Cu^2+^ concentration were studied. For each experimental run, 100 mL of Cu^2+^ solution of known concentration was taken in a 250 mL stoppered reagent bottle. The solution was stirred by a magnetic stirrer continuously during the adsorption experiment. About 20 mg of adsorbent was used in all experiments. The Cu^2+^ uptake *q* (mmol·g^−1^) was determined by the following equation:

(2) $$q=\frac{\left(C_{0}-C_{t}\right)\times V}{m}$$

where *C*_0_ and *C*_t_ are the initial and final Cu^2+^ concentrations (mmol·L^−1^), respectively; *V* is the volume of solution (L), and *m* is the mass (g) of adsorbent used. The Cu^2+^ concentration of solution was determined by atomic adsorption spectrophotometer (Varian Spectra HP 3510, Santa Clara, CA, USA).

### 2.5. Desorption Experiments

After being immersed into Cu^2+^ solution (1 mmol·L^−1^, pH 5.3) for 5 h, the adsorbent was taken out from the Cu^2+^ solution. The adsorbed Cu^2+^ was removed by stirring the adsorbent in 10 mL of HCl (0.1 mol·L^−1^) solution for 30 min. After thorough washing, they were reused for the next new Cu^2+^ solution (100 mL, 1 mmol·L^−1^). The adsorption-desorption test of hybrid adsorbent was repeated 10 consecutive times.

### 2.6. Model to Experimental Data

For adsorption kinetics study, two models—pseudo-first-order and pseudo-second-order—were employed to analyze the experimental data.

The pseudo-first-order model is described as [2]:

(3) $$q_{t}=q_{e}-q_{e}e^{-K_{1}t},$$

where *K*_1_ is the pseudo-first-order rate constant (min^−1^); *q_e_* and *q_t_* are the metal ion uptake (mmol·g^−1^) at equilibrium and at time *t*, respectively.

The pseudo-second-order model is described as [2]:

(4) $$\frac{t}{q_{t}}=\frac{1}{K_{2}q_{e}^{2}}+\left(\frac{1}{q_{e}}\right)t$$

where *K*_2_ is the pseudo-second-order rate constant (g·mmol^−1^·min^−1^).

For adsorption isotherm study, two models—Langmuir and Freundlich—were employed to analyze the experimental data.

The Langmuir isotherm equation is given below [2]:

(5) $$q_{e}=\frac{bq_{max}C_{e}}{1+bC_{e}},$$

where *C*_e_ is the concentration of heavy metal ion solution at equilibrium (mg·L^−1^); *q*_max_ signifies the adsorption capacity (mg·g^−1^) and *b* is related to the energy of adsorption.

The Freundlich isotherm equation is given below [2]:

(6) $$q_{e}=K_{f}C_{e}^{n},$$

where *K*_f_ (mg·g^−1^) represents adsorption capacity and *n* is related to adsorption intensity.

## 3. Results and Discussion

### 3.1. Preparation and Characterization of Adsorbents

#### 3.1.1. Effects of AMPS and Chitosan Concentrations on the Dispersion Property of Laponite

**Figure 1:**
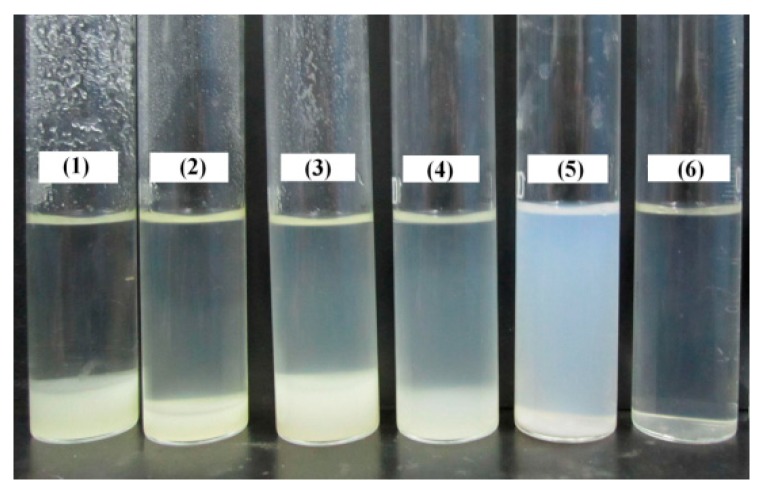
Effect of AMPS concentration on the dispersion property of Laponite in chitosan solution. Concentrations: Laponite 0.5 wt %; chitosan 1 wt %; AA 0.4 wt %; AMPS (**1**) 0 wt %; (**2**) 0.5 wt %; (**3**) 1 wt %; (**4**) 1.5 wt %; (**5**) 2 wt %; (**6**) 2.5 wt %.

**Figure 2:**
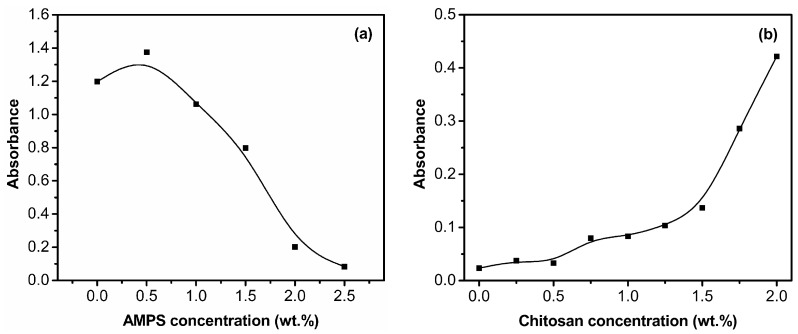
Effects of AMPS concentration (**a**) and chitosan concentration (**b**) on the absorbance value of Laponite dispersion system at 680 nm. Concentrations: (**a**) Laponite 0.5 wt %, chitosan 1 wt %, AA 0.4 wt %; (**b**) Laponite 0.5 wt %, AMPS 2.5 wt %, AA 0.4 wt %.

Chitosan is only soluble in diluted mineral and organic acids, except for sulfuric acid because of the protonation of its amine groups. However, Laponite nanoparticles will aggregate in acidic or polymer solution. As a result, in order to improve the dispersion property of Laponite nanoparticles in acidic chitosan solution, they can be adsorbed by AMPS molecules before AA and acidic chitosan solution are added into the system. The AMPS concentration has a great influence on the dispersion property of Laponite nanoparticles as shown in Figure 1. When the AMPS concentration is less than 1.5 wt %, obvious flocculation phenomena are observed. When the AMPS concentration is more than 2.0 wt %, the bottom layer flocculate disappears and a uniform system is obtained. The absorbance values of different dispersion systems were measured as shown in Figure 2. It is indicated that, when the AMPS concentration is 2.5 wt %, Laponite can disperse as nanoscale particles in a broad concentration range of chitosan (from 0 to 1.5 wt %). In our experiment, four samples with different ratios of chitosan/Laponite/monomers—named S-1, S-2, S-3, and S-4—were prepared as presented in Table 1. S-1 is free of Laponite and chitosan, S-2 is free of Laponite and S-3 is free of chitosan. For S-3 and S-4, the concentrations of AMPS and chitosan are suitable for the favorable dispersion property of Laponite nanoparticles.

#### 3.1.2. FTIR Analysis of Four Samples

**Figure 3:**
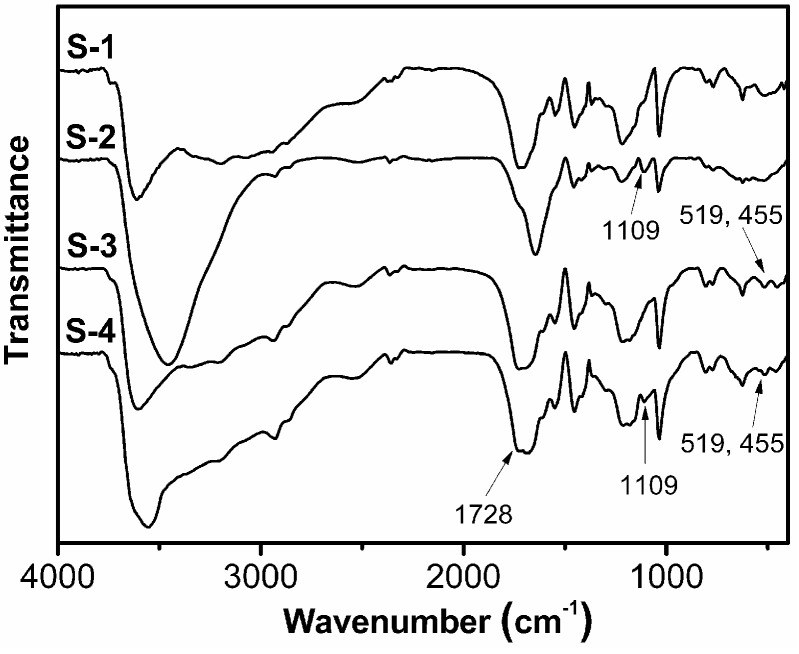
FTIR spectra of S-1, S-2, S-3, and S-4.

The structures of S-1, S-2, S-3, and S-4 were evaluated by FTIR as shown in Figure 3. The presence of chitosan component in S-2 and S-4 is indicated by the peak at 1109 cm^−1^, which is classically assigned to the polysaccharide molecule backbone vibrations, and the peaks at 519 and 455 cm^−1^ for S-3 and S-4 are assigned to the absorption band of Si-O in Laponite. The peaks around 1600 cm^−1^ are assigned to the absorption band of the carbonyl groups of AM, AMPS, or chitosan. Moreover, carbonyl groups of carboxylic acids usually absorb at a high wavenumber (1800–1650 cm^−1^) if they are present in an acidic form. The appearing in the spectrum of four samples at 1728 cm^−1^ within this spectral region is consistent with the preparation (acidic conditions). The broad peaks around 3500 cm^−1^ of four samples can be related to the absorption band of O–H or N–H from different functional groups.

#### 3.1.3. SEM Analysis of Four Samples

**Figure 4:**
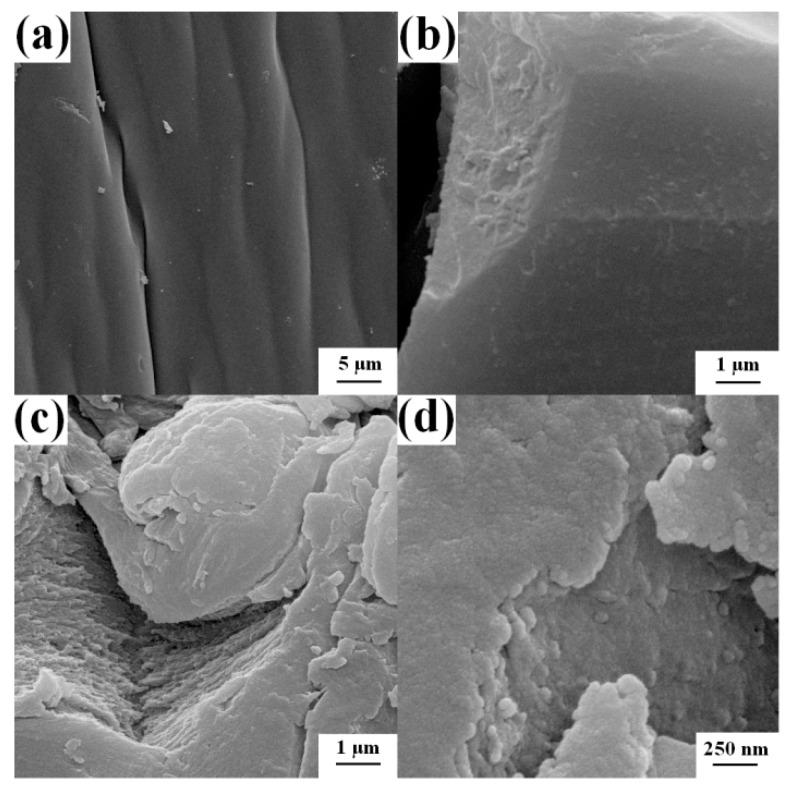
SEM images of S-2 (**a**,**b**) and S-4 (**c**,**d**).

The surface morphologies of S-2 and S-4 were observed with SEM as shown in Figure 4. S-2 shows a corrugated surface, and S-4 represents a more uneven surface. Moreover, there are many micropores and small aggregates on the surface of S-4, which might be convenient for the penetration of metal ions into the network and thus enhance its adsorption ability. The irregular granular structures with the size of 50–200 nm might be formed by the aggregation of Laponite nanoparticles. The element component on the surface of S-4 was obtained from EDS analysis. Two points were measured and the result was shown in Table 2. In these two points, the high atomic percentages of carbon, nitrogen, oxygen, and sulfur elements represent the high content of polymer components. Moreover, these are both low atomic percentages of magnesium and silicon elements in these two points, which indicates the component of Laponite in the sample.

#### 3.1.4. BET Analysis of Four Samples

**Figure 5:**
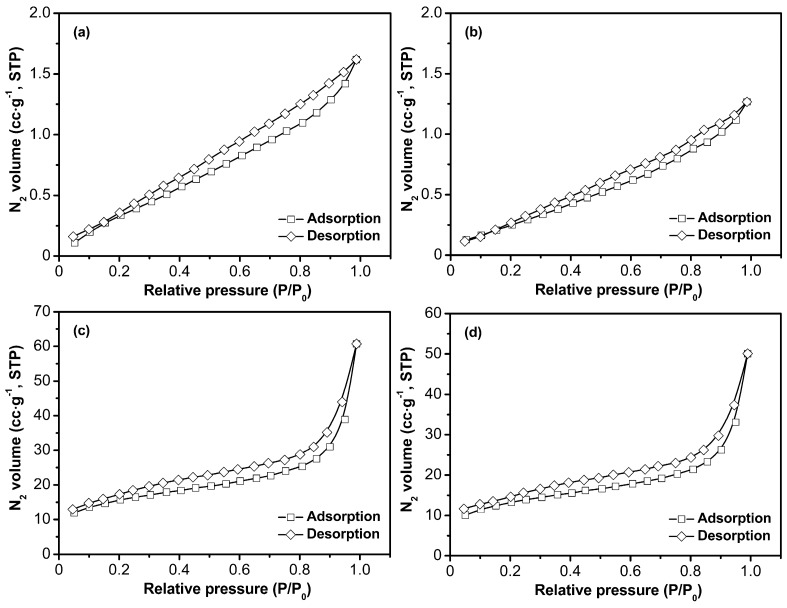
N_2_ adsorption-desorption isotherms of S-1 (**a**), S-2 (**b**), S-3 (**c**), and S-4 (**d**).

**Figure 6:**
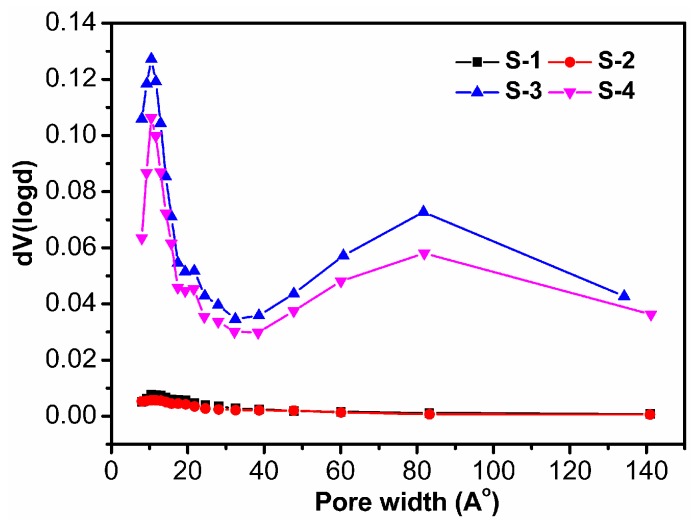
Pore size distribution curves of four samples.

The incorporation of Laponite nanoparticles within the polymer matrix was expected to improve its surface properties, such as its surface area and pore diameter. Therefore, the structural differences in these four samples were studied via BET analysis and the results are shown in Table 3, Figure 5 and Figure 6. For S-2, the BET surface area and pore volume are 1.19 m^2^·g^−1^ and 0.00195 cm^3^·g^−1^, respectively. However, after nanocomposite formation with Laponite particles, the surface area and pore volume for S-4 are 44.69 m^2^·g^−1^ and 0.07715 cm^3^·g^−1^, respectively. From the result of pore size distribution analysis as shown in Figure 6, it is indicated that the main population of pores occurred at about 1 nm and 8 nm, and the pore size distribution between 1 and 14 nm appears to be continuous. Herein, the in situ incorporation of Laponite nanoparticles onto the polymer matrix enhances the surface area, average pore diameter, and total pore volume. This experimental result reflects that the interfacial interaction between the polymer and nano filler significantly affects the pore structure of the hybrid composite material. Similar changes in the surface properties of polymers after the incorporation of nanoparticles have also been reported [41,42].

### 3.2. Adsorption Studies

#### 3.2.1. Adsorption Kinetics

**Figure 7:**
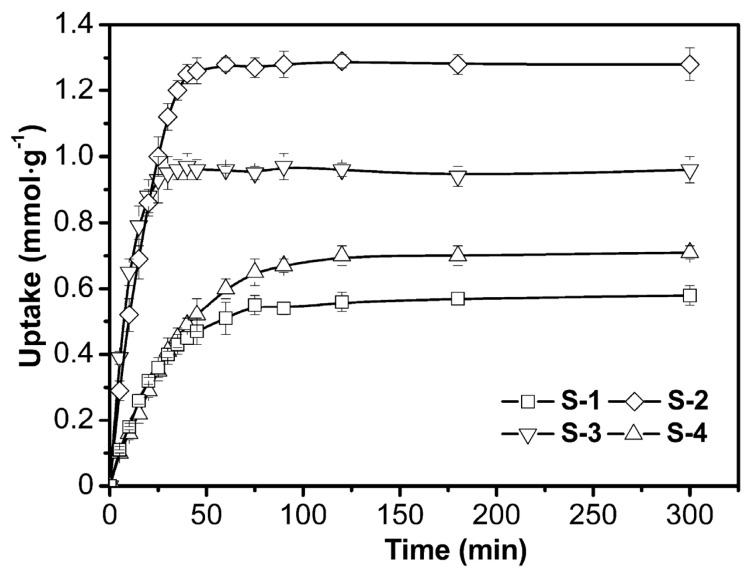
Adsorption kinetics of Cu^2+^ by different adsorbents (30 °C; pH 5.3; initial Cu^2+^ concentration 1 mmol·L^−1^).

The removal capacities of four adsorbents for Cu^2+^ as a function of time were studies as shown in Figure 7. The removal of Cu^2+^ occurs in the order of S-4 (1.28 mmol·g^−1^) > S-3 (0.96 mmol·g^−1^) > S-2 (0.71 mmol·g^−1^) > S-1 (0.58 mmol·g^−1^). From the results, it is indicated that this hybrid adsorbent has a good performance for Cu^2+^ removal. It is also indicated that the addition of chitosan and Laponite could greatly increase the adsorption capacity of adsorbent. Additionally, these adsorbents containing a Laponite component (S-3 and S-4) possess the advantage of quick adsorption. It takes less than 35 min for S-3 and S-4 until an equilibrium adsorption state is reached. However, the time is more than 75 min for S-1 and S-2.

Adsorption kinetics is an important step for the understanding of adsorption mechanisms and evaluation of adsorbent performance. Pseudo-first-order and pseudo-second-order kinetics models are two widely used models to analyze the solid-liquid adsorption. A very high correlation between experimental data and two kinetics models (*R*^2^ > 0.988) are obtained as shown in Table 4. The numerical result of *K*_1_ (pseudo-first-order model) is in the order of S-3 (0.113) > S-4 (0.057) > S-1 (0.040) > S-2 (0.028), and it was in the order of S-3 (0.523) > S-4 (0.121) > S-1 (0.087) > S-2 (0.042) for *K*_2_ (pseudo-second-order model). Thus it can be seen that Laponite nanoparticles obviously improve the adsorption rate of these adsorbents.

#### 3.2.2. Effect of pH

**Figure 8:**
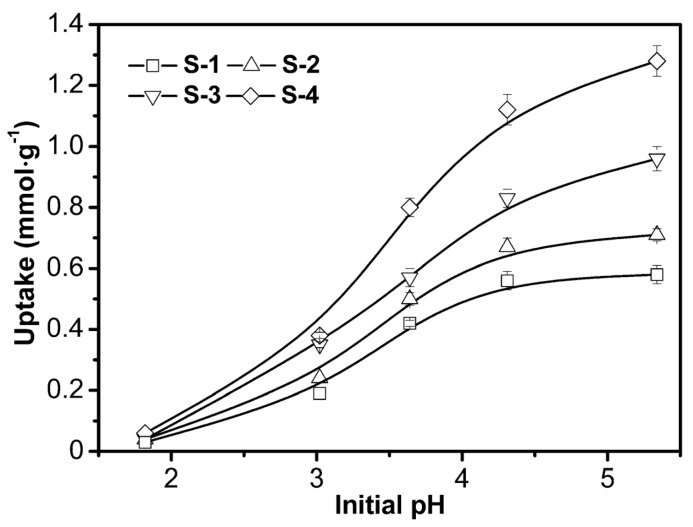
Effects of initial pH on the adsorption capacities of four samples. Conditions: initial Cu^2+^ concentration 1 mmol·L^−1^, 30 °C.

The solution pH is an important parameter influencing the adsorption process at the water-sorbent interface. To determine the optimum pH for the maximum removal of Cu^2+^ by four samples, the equilibrium adsorption of Cu^2+^ was investigated over a pH range of 1.8–5.3 as shown in Figure 8. At low initial pH, the adsorption capacities for all adsorbents are insignificant and when it increases from 1.8 to 4.3, sharp increases of adsorption capacities are observed. This might be mainly due to the dissociation of functional groups in polymer and the charge change on the Laponite surface. Previous research has shown that, at low pH values, the concentration of H^+^ can alter the speciation of some active functionalities such as carboxylate and amine, protonating them to carboxylic acid and ammonium, which reduces the adsorption [1,27,31]. When initial pH increases from 4.3 to 5.3, there are no remarkable differences in the adsorption capacities for S-1 and S-2. However, for S-3 and S-4, especially for S-4, the adsorption capacity increases obviously when the pH increases from 4.3 to 5.3. Due to hydroxyl groups, oxygen atoms and negative charges on the Laponite surface, there is interaction between Laponite nanoparticles and other components. The pH change might affect this interaction and Laponite nanoparticles might be sensitive to pH.

**Figure 9:**
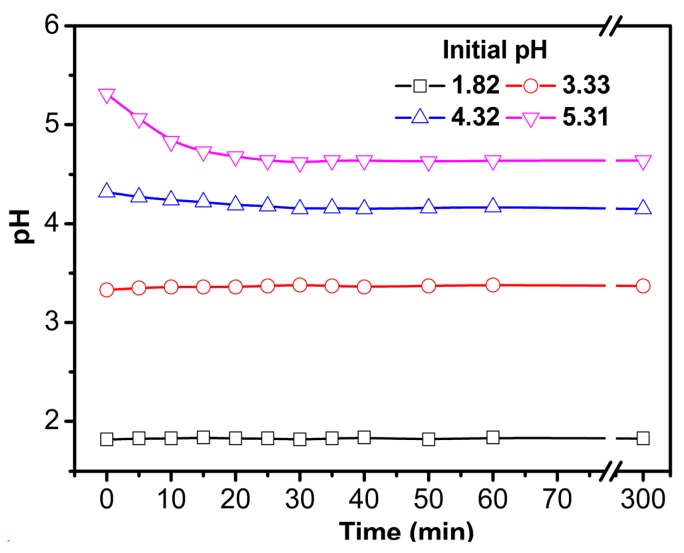
pH change in adsorption process for S-4 (initial Cu^2+^ concentration, 1 mmol/L; temperature, 30 °C).

In the adsorption test, pH change was observed as shown in Figure 9. When the pH value is 1.8 or 3.3, it changes a little in the whole adsorption test. When the pH value is 5.3, it decreases a lot in the beginning stage of adsorption. After 20 min, the pH value obtains a balanced value (4.68). The pH change might be due to the dissociation of ionic functional groups and ion exchange. In addition, the decrease of pH might affect the actual adsorption capacity of this material.

#### 3.2.3. Effect of Temperature

**Figure 10:**
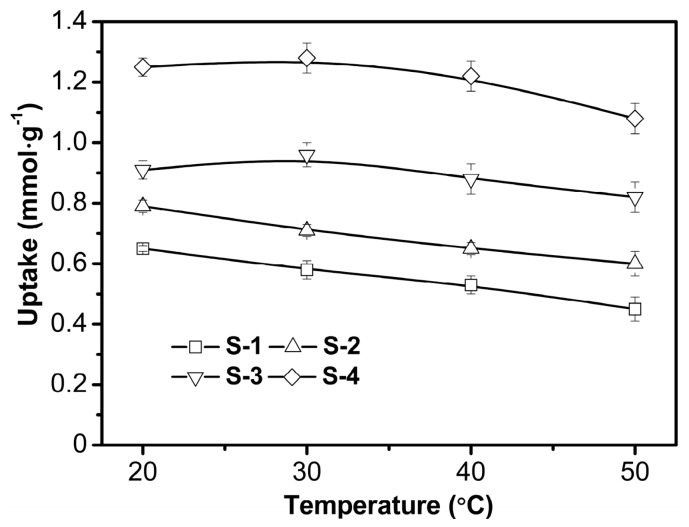
Effects of temperature on the adsorption capacities of four samples. Conditions: initial Cu^2+^ concentration 1 mmol·L^−1^, pH 5.3.

The temperature has two major effects on the adsorption process. Temperature dependence of the adsorption system dictates the adsorption as endothermic or exothermic. Increasing the temperature is recognized to increase the rate of diffusion of the adsorbate, owing to the decrease in the viscosity of the solution [43,44]. In this experiment, the temperature was set at 20–50 °C according to the usual temperature regime in wastewater treatment. As shown in Figure 10, slight increase in the adsorption efficiency was observed for S-3 and S-4 upon increasing the temperature from 20 to 30 °C. Further temperature increases led to minor decreases in the adsorption capacity. The adsorption capacity for S-1 and S-2 decreased all the way from 20 to 60 °C. An increase in temperature may facilitate the transfer of metal ions from aqueous solution into adsorbent, however, higher temperature may also induce a desorption process of metal ions [1].

#### 3.2.4. Adsorption Isotherm

**Figure 11:**
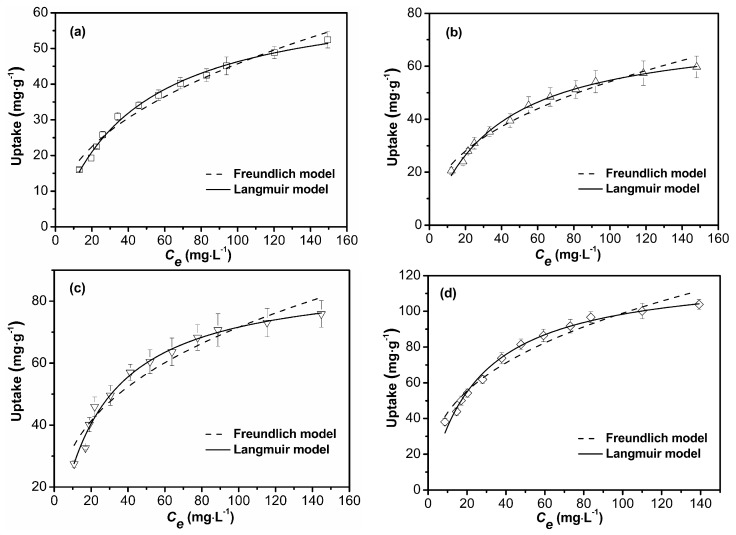
Adsorption isotherm of Cu^2+^ by S-1 (**a**), S-2 (**b**), S-3 (**c**), and S-4 (**d**) (30 °C; pH 5.3).

In the adsorption process, the distribution of solute between the solid and liquid phase and measurement of the distribution coefficient can be studied by utilizing various adsorption isotherms [25]. In order to disclose the nature of the interaction between the adsorbent and adsorbate molecules, the Langmuir and Freundlich isotherm models were employed to fit the Cu^2+^ removal process by four samples as shown in Figure 11 and Table 5.

Langmuir isotherm describes the formation of a monolayer adsorption with physical interaction. The basic theory for it is that all the adsorption sites on the adsorbent surface are homogeneous, having equal affinity to the adsorbate molecules [45]. Freundlich isotherm is normally used to describe multilayer adsorption occurring on the heterogeneous surface of adsorbents. The basic theory for it is that the distribution of adsorption sites on the adsorbent surface are heterogeneous, the stronger adsorption sites are occupied first, and with an increase in the coverage of adsorbent surface, the affinity of adsorbent to adsorb decreases [46]. In the present study, according to the correlation coefficient, it could be concluded that the Langmuir model provided a better fit than Freundlich model to the adsorption process for all four adsorbents. This suggests that adsorption occurs on the structurally homogeneous surface of adsorbent with identical binding sites. The parameter of *n* from Freundlich model for S-3 and S-4 is smaller than that for S-1 and S-2, reflecting the extent of the bond strength between adsorbate and adsorbent due to the addition of Laponite in the adsorbent [1].

### 3.3. Desorption Studies

**Figure 12:**
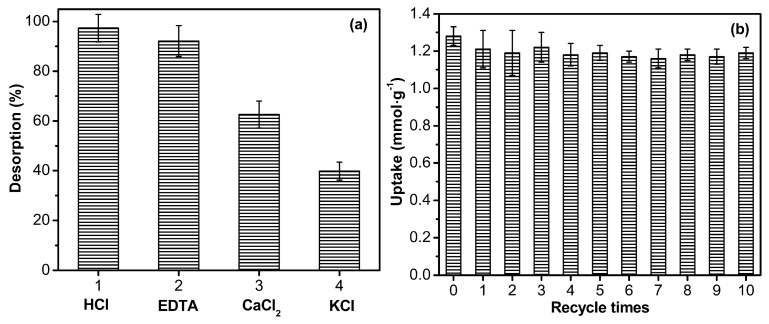
The regeneration and recycle of S-4: (**a**) the effect of eluting chemical on the desorption rate (acid or salt concentration 0.1 mol·L^−1^) and (**b**) recyclable adsorption of Cu^2+^ by S-4.

Reusability is a very important requirement for adsorbent, which will reduce the material-cost significantly in practical application. To evaluate the property of regeneration of nanocomposite adsorbent, various regents—such as HCl, EDTA, KCl, and CaCl_2_—were tested as eluents for S-4 as shown in Figure 12a. Cu^2+^ desorption is almost complete (desorption percentage, 97.3%) when the loaded adsorbent was treated with 0.1 mol·L^−1^ HCl, but when it is treated with 0.1 mol·L^−1^ KCl, the desorption percentage was only 39.8%. Thus, HCl was selected for the regeneration of S-4. As shown in Figure 12b, there is a small loss of adsorption capacity after the first reuse cycle. However, the adsorption capacities do not show any significant decrease after the second reuse cycle. As the reusability experiments were conducted in sequence for 10 runs, the equilibrium adsorption capacity decreases from 1.28 mmol·g^−1^ to 1.19 mmol·g^−1^, an excellent adsorption capacity, which is 93.0% of the initial adsorption capacity.

## 4. Conclusions

Novel chitosan and Laponite-based nanoparticle-polymer hybrid material was prepared and it was demonstrated to be a rapid and effective adsorbent for the removal of Cu^2+^ from aqueous solution. Herein, the addition of chitosan and AMPS-stabilized Laponite nanoparticles into adsorbent is essential for the improvement of adsorption rate and capacity. Additionally, excellent regenerative efficacy of the hybrid adsorbent was obtained after soaking in 0.1 mol·L^−1^ HCl solution for 30 min, which will improve the economic benefit of the process.

## Figures and Tables

**Table 1:** The feeding composition of four samples.

Sample	AM (g)	AA (g)	AMPS (g)	MBA (g)	H_2_O (g)	Laponite (g)	Chitosan (g)	KPS (g)	Yield (%)
S-1	5.0	2.5	2.5	0.03	90.0	0	0	0.05	96.9
S-2	5.0	2.5	2.5	0.03	90.0	0	0.5	0.05	96.3
S-3	5.0	2.5	2.5	0.03	90.0	0.5	0	0.05	98.1
S-4	5.0	2.5	2.5	0.03	90.0	0.5	0.5	0.05	98.6

**Table 2:** The element components of S-4 surface for EDS analysis.

Element	Atomic %	Atomic %
C	55.30	56.04
N	11.63	11.55
O	29.91	28.42
S	1.72	1.98
Mg	0.61	0.85
Si	0.83	1.16
Total	100.00	100.00

**Table 3:** Structural characteristics of four samples.

Sample	*A*_BET_ (m^2^·g^−1^)	*V*_total_ (cm^3^·g^−1^)	*D* (nm)
S-1	1.89	0.00249	5.27
S-2	1.19	0.00195	6.55
S-3	52.86	0.09350	7.08
S-4	44.69	0.07715	6.90

**Table 4:** Adsorption kinetics constants for Cu^2+^ adsorbed by different adsorbents.

Model	Coefficient	S-1	S-2	S-3	S-4
Pseudo-first	*K*_1_	0.040	0.028	0.113	0.057
	*q*_e_	0.57	0.72	0.97	1.31
	*R^2^*	0.9985	0.9967	0.9970	0.9886
Pseudo-second	*K*_2_	0.087	0.042	0.523	0.121
	*q*_e_	0.62	0.81	0.98	1.34
	*R*^2^	0.9970	0.9886	0.9991	0.9953

**Table 5:** Adsorption isotherm constants for Cu^2+^ adsorbed by different adsorbents.

Model	Coefficient	S-1	S-2	S-3	S-4
Langmuir	*q*_max_	66.16	74.82	88.85	122.76
	*b*	0.023	0.027	0.042	0.040
	*R*^2^	0.9936	0.9947	0.9874	0.9892
Freundlich	*K*_f_	5.99	8.22	14.91	18.88
	*n*	0.44	0.41	0.34	0.36
	*R*^2^	0.9741	0.9709	0.9359	0.9614
